# Out-of-pocket expenditures for childbirth in the context of the Janani Suraksha Yojana (JSY) cash transfer program to promote facility births: who pays and how much? Studies from Madhya Pradesh, India

**DOI:** 10.1186/s12939-016-0362-4

**Published:** 2016-05-03

**Authors:** Kristi Sidney, Mariano Salazar, Gaetano Marrone, Vishal Diwan, Ayesha DeCosta, Lars Lindholm

**Affiliations:** Department of Public Health Sciences, Karolinska Institutet, Widerströmska, Tomtebodavägen 18A, plan 4, SE-171 77 Stockholm, Sweden; Public Health and Environment, R.D. Gardi Medical College, Ujjain, Madhya Pradesh India; International Center for Health Research, R.D. Gardi Medical College, Ujjain, Madhya Pradesh India; Department of Public Health and Clinical Medicine, Umeå University, Umeå, Sweden

**Keywords:** Out-of-pocket expenditure, Conditional cash transfer, Maternal health, Facility based delivery, India

## Abstract

**Background:**

High out-of-pocket expenditures (OOPE) make delivery care difficult to access for a large proportion of India’s population. Given that home deliveries increase the risk of maternal mortality, in 2005 the Indian Government implemented the Janani Suraksha Yojana (JSY) program to incentivize poor women to deliver in public health facilities by providing a cash transfer upon discharge. We study the OOPE among JSY beneficiaries and women who deliver at home, and predictors of OOPE in two districts of Madhya Pradesh.

**Methods:**

September 2013 to April 2015 a cross-sectional community-based survey was performed. All recently delivered women were interviewed to elicit delivery costs, socio-demographic characteristics and delivery related information.

**Results:**

Most women (*n* = 1995, 84 %) delivered in JSY public health facility, the remaining 16 % (*n* = 386) delivered at home. Women who delivered under JSY program had a higher median, IQR OOPE ($8, 3–18) compared to home ($6, 2–13). Among JSY beneficiaries, poorest women had twice net gain ($20) versus wealthiest ($10) post cash transfer. Informal payments (64 %) and food/baby items (77 %) were the two most common sources of OOPE. OOPE made among JSY beneficiaries was pro-poor: poorer women made proportionally less expenditures compared to wealthier women. In an adjusted model, delivering in a JSY public facility increased odds of incurring expenditures (OR: 1.58, 95 % CI: 1.11–2.25) but at the same time to a 16 % (95 % CI: 0.73–0.96) decrease in the amount paid compared to home deliveries.

**Conclusions:**

OOPE is prevalent among JSY beneficiaries as well in home deliveries. In JSY, OOPE varies by income quintile: wealthier quintiles pay more OOPE. However the cash incentive is adequate enough to provide a net gain for all quintiles. OOPE was largely due to indirect costs and not direct medical payments. The program seems to be effective in providing financial protection for the most vulnerable groups.

## Background

India’s health care services are largely financed by out-of-pocket payments (71 %) at the point of care [1]. High out-of-pocket expenditures (OOPE) make health services, including care for childbirth, difficult to access for a large proportion of its population especially the poor. In 2005, institutional delivery was nearly 6.5 times higher among Indian women belonging to the highest wealth quintile (84 %) compared to poorest quintile women (13 %) [2].

Evidence suggests maternal mortality can be reduced when deliveries are conducted by skilled birth attendants and women have access to emergency obstetric care, given the unpredictable nature of life-threatening complications that can occur at the time of childbirth [3]. Poor women, who have the least access to such care, bear a disproportionate burden of maternal mortality.

Therefore governments in many low income settings, with high burdens of maternal mortality, have initiated special programs to draw women into facilities to give birth (instead of at home), where such care can be provided. Given the high number of maternal deaths in India (one-fifth of the global count) and a low institutional delivery proportion (39 % in 2005); the Indian government launched a conditional cash transfer program to promote institutional delivery among poor women the same year [2, 4]. The program, Janani Suraksha Yojana (JSY or safe motherhood program), provides women $23 (INR 1400) upon discharge after giving birth in a public health facility.

JSY, the largest cash transfer program in the world, is funded by the central government of India (GOI) while implementation is managed by states. Eligibility, incentive amount and uptake differ across the states [5, 6]. In the ten years since the program began, institutional delivery rates have increased to 74 % and more than 106 million women have benefited with the GOI spending 16.4 billion USD on the program [7–9].

Previous studies have found a considerable proportion of Indian women cite financial access barriers as one of the main reasons for not having an institutional delivery [10–12]. The JSY program was expected to draw these mothers into public facilities with the assumption that they will receive a free delivery in addition to receiving the cash incentive of $23. While all services in the Indian public sector are supposed to be free, in reality OOPE during childbirth is common among these facilities [13–17].

Although there are a number of reports on the JSY [18–22], none focus on OOPE in the context of high JSY program uptake [13–17, 23]. The magnitude of OOPE incurred among these JSY beneficiaries is unknown. In addition, we do not know if the cash incentive offsets OOPE in the same facility, and if it does then the extent to which it does. Also the question remains whether the level of OOPE paid by JSY beneficiaries of different socio-economic status is similar. Further, if women who participate in the JSY program actually have higher OOPE than women who give birth at home. We studied the OOPE among JSY beneficiaries and compared this OOPE to that incurred by women who delivered at home in two districts of Madhya Pradesh, India. We also described the extent to which the JSY cash transfer defrayed the OOPE for JSY beneficiaries. Among both groups of women, we studied predictors of OOPE, and how OOPE varied with wealth status.

## Methods

### Study area

The study took place in two of the 51 administrative districts that make up Madhya Pradesh (MP), a state in central India. With a population of 71 million, it is one of India’s largest states [24]. MP on the whole has poorer socio-economic and health indicators relative to country (India) averages; 24 % of the population lives below the poverty line and has a maternal mortality ratio (MMR) of 190 per 100,000 live births [25]. In MP, all women are eligible to be beneficiaries of the JSY program regardless of their poverty status, if they deliver in a public health facility. MP has had one of the highest uptakes of JSY in the country [26].

The two study districts were purposively selected to represent different socio-economic and geographic areas of MP. They were also study areas of a larger project (MATIND) [27] to study the JSY program in which this study was nested. Each study district (population 1.5–2 million) is divided into 5 administrative blocks (100,000–200,000 people per block), each block comprises of villages with an approximate population of 1000–10,000 people per village. District one, situated on the western side of the state, has relatively better socio-economic characteristics, a lower MMR (176) and a higher uptake of the JSY program (72 %) [26]. In comparison, district two which lies on the eastern side of the state, has generally poorer socio-economic characteristics, a higher MMR (361), and a lower JSY uptake (59 %) [26]. The districts not only differ on socio-economic characteristics, but also in regards to geographical features. The first district is relatively more urban and has a flat geographical terrain, while the second district is mostly rural, has several hill ranges as well as dense forested areas.

### Study design and sampling

A community-based cross-sectional study was conducted from September 2013 to April 2015. Study villages were selected through a combination of multi-stage sampling in the two districts. In the first district, all blocks were included. In the second district, two out of the five blocks were purposively selected, one in the north and the other in the south of the district. Within the selected blocks, villages that had between 200 and 10,000 inhabitants were included in the sampling frame. All villages were stratified into two groups based on a five kilometer (km) distance from a public health facility that conducted more than 10 deliveries in a month. Probability proportionate to size was used to select the individual study villages ensuring the number of sample units in each stratum would be allocated in proportion to their share in total population. In total, 247 villages were selected, 101 and 146 villages within and outside the five km radius of a facility, respectively.

### Data collection

The study participants (i.e., pregnant women who had just given birth in each village) were identified with the help of local community health workers (Accredited Social Health Activist), local crèche workers, and traditional birth attendants (*Dai*). The community workers were incentivized to report births to the research team within two days of delivery. As an additional measure to ensure all births were identified, research teams frequently visited the study villages throughout the recruitment period. Trained research assistants interviewed recently delivered mothers at a health facility or in their home within the first week of delivery. They elicited information on out-of-pocket expenditures (OOPE) for the current childbirth and other details including maternal socio-demographic characteristics, birth order, household wealth, location of delivery, and type of delivery. Family members provided additional information on the costs if the mother could not recall or did not know.

During the recruitment period, 2779 births were reported and 2615 women were enrolled in the study. The reasons given for not enrolling in the study were (i) the mother had migrated back to her resident village (*n* = 108), (ii) the home was not accessible to the research team (*n* = 52), (iii) maternal death (*n* = 8) and (iv) two women refused to participate. A small number of women (*n* = 8) reported that they did not know the cost of the delivery thus were excluded from the analysis. Women who delivered in a private facility (*n* = 226) were removed from the analysis as the study focused on the JSY program and OOPE. Also these women have significantly different characteristics; they tend to be wealthier, live in urban settings and are not the primary focus of the JSY program.

### Definitions and variables used

The main outcome of interest, OOPE, was a sum of the following expenditures (i.e., gross OOPE): 1. *Medicine, supplies and procedures* (i.e., delivery costs, medicines, supplies, blood transfusions, diagnostic tests, and anesthesia); 2. *Informal payments* (i.e., expenditures reported as ‘rewards’ paid by the women/families to the staff for assisting their care in the facility. For the women who delivered at home, cash given to the *dai* (traditional birth attendant who conducts home deliveries) was classified as an informal payment; 3. *Food/Cloth* (i.e., food consumed during hospital stay or at home in relation to the delivery and cloths used for the infant), and 4. *Transportation costs* (i.e., all costs for the mother and her attendants associated with reaching the health facility for delivery). The OOPE were collected in Indian rupees (INR) and converted to U.S. dollars (US$) using the exchange rate at the time of the study of 60 INR to US$1.

Independent variables: Socio-demographic characteristics included age and education as continuous variables and caste^1^ as a categorical variable. Birth order was categorized by number of live births up to four (or more). Women’s household wealth was assessed by using a standard technique involving principal component analysis to construct a wealth index based on socio-economic characteristics developed for the standard of living index in the National Family Health Survey. The variables included 20 household assets, structural material of dwelling, sanitation provisions, and land ownership [28, 29]. The household wealth index was calculated for the entire study sample, then the score was categorized into five quintiles. We used the wealth index as a proxy measurement for wealth as income is a difficult variable to collect in low-income settings. The mother could have delivered under the JSY program at public facility or at home. JSY beneficiaries were any woman who delivered in a public health facility and thus eligible to receive the cash incentive. Type of delivery was classified into vaginal or cesarean section. We define the ‘net gain’ as the JSY incentive ($23) minus the OOPE.

### Analysis

Univariate, bivariate and multivariable statistics were used to describe and analyze the data. The dependent variable, OOPE, was not normally distributed thus median and interquartile range (IQR) were used to describe the data. Wilcoxon-Mann-Whitney and Kruskal Wallis tests were used to compare OOPE differences between groups.

Inequalities in OOPE were analyzed using the concentration curve and concentration index as demonstrated by Wagstaff et al. [30]. The concentration curve plots the cumulative percentage of OOPE (y-axis) against the cumulative percentage of the women, ranked by their wealth index, from the poorest to the richest households (x-axis) [31]. We used the concetration curve to graphically display the inequalities in OOPE in this population. The concentration index (CI), a range of −1 to 1, allowed us to quantify the inequality in the OOPE. CI is defined graphically as twice the area between the concentration curve and the line of equality [32]. A positive value indicates a progressive system where the wealthy have more proportion of OOPE compared to the poor, while a negative value indicates the opposite (and a regressive system).

### Multivariable analysis

A two-part model, developed as part of the Rand Health Insurance Experiment, was used for the multivariable regression to assess determinants of OOPE [33–36]. This model is commonly used when studying health expenditures to accommodate the significant number of zeros (no expense incurred) and for its distribution (i.e., right skewed with a long tail) which fits our data. The first part, a binary logistic model, was used to understand the predictors associated with any OOPE and estimated the odds of a woman incurring any OOPE. We present the coefficients as adjusted odds ratios (AOR) and 95 % Confidence Intervals.

The second part of the model, a generalized linear model (GLM) with a negative binomial distribution, analyzed the determinants of OOPE among women who reported *any* OOPE. This model estimated the OOPE for women who reported incurring an expense. We adjusted for observable characteristics that may influence delivery-related OOPE based on previous literature and context specific knowledge. We present the coefficients as incident rate ratios (IRR) and 95 % Confidence Intervals.

Multicollinearity was assessed by calculating the mean variance inflation factor (VIF = 1.53), which did not show evidence of collinearity. No interaction existed between the variables in the model. In all the models, *p*-values <0.05 were considered significant.

### Ethical considerations

The study was described to all study participants. Informed consent was obtained from the participants before they were enrolled in the study and responded to the questionnaire. Anonymity and confidentiality was ensured to all women. Ethical approval for the study was granted by the Ethics Committee from the authors’ institution.

## Results

### Descriptive sample characteristics for the sample

As depicted in Table 1, the majority of women (*n* = 1995, 84 %) in our study delivered in a JSY public health facility. The remaining 16 % (*n* = 386) delivered at home. The median age of the study sample was 23 years and 29 % (*n* = 692) had no formal education. More than a third (*n* = 932) of the women were primiparous. The main reason given for home deliveries was that the baby came unexpectedly and quickly (*n* = 312, 52 %). Other reasons included; planned to have a home delivery (*n* = 97, 16 %), transportation related issues (*n* = 95, 16 %), no one to accompany them to the hospital (*n* = 58, 10 %) and other (*n* = 37, 6 %). Only one woman replied she could not afford to deliver in a health facility (results not shown).

**Table 1:** Background characteristics, median and inter-quartile range (IQR) of gross OOPE (in U.S. dollars) for women who delivered in a JSY facility or at home. Column percentages (%)

	All women		JSY beneficiary		Home delivery	
Background characteristics	n (%)	Median (IQR)	n (%)	Median (IQR)	n (%)	Median (IQR)
Total	2381 (100)	8 (3–17)	1995 (84)	8 (3–18)	386 (16)	6 (2–13)
Age in years (median, IQR)	23 (21–25)	–	22 (20–25)	–	25 (22–27)	–
Districts						
District 1	1405 (59)	14 (7–23)*	1251 (63)	14 (7–23)*	154 (40)	12 (5–23)*
District 2	976 (41)	3 (1–7)	744 (37)	3 (1–7)	232 (60)	3 (1–7)
Education in years (median, IQR)	5 (0–8)	–	5 (0–8)	–	4 (0–7)	–
Household wealth						
1st quintile (Poorest)	519 (22)	3 (1–7)**	361 (18)	3 (1–6)**	158 (40)	3 (0–7)**
2nd quintile	505 (21)	7 (2–13)	414 (21)	6 (2–15)	91 (24)	7 (2–12)
3rd quintile	499 (21)	11 (5–18)	434 (22)	11 (5–18)	65 (17)	10 (3–18)
4th quintile	476 (20)	14 (7–23)	429 (21)	14 (7–23)	47 (12)	8 (3–19)
5th quintile (Least Poor)	382 (16)	13 (7–25)	357 (18)	13 (7–24)	25 (7)	17 (8–29)
Caste						
Scheduled Caste (SC)	599 (25)	10 (4–19)**	502 (25)	11 (5–20)**	97 (25)	8 (2–17)**
Other backward caste (OBC)	904 (38)	12 (5–22)	807 (40)	12 (5–22)	97 (25)	8 (4–17)
Scheduled Tribe (ST)	598 (25)	3 (1–6)	430 (22)	3 (1–6)	168 (44)	3 (1–7)
General	280 (12)	13 (4–21)	256 (13)	12 (4–22)	24 (6)	16 (5–20)
Birth Order						
1st child	932 (39)	8 (3–19)**	858 (43)	9 (3–20)**	74 (19)	7 (3–12)**
2nd child	838 (35)	8 (3–17)	688 (34)	9 (3–17)	150 (39)	7 (2–15)
3rd child	382 (16)	5 (2–14)	292 (15)	7 (2–15)	90 (23)	4 (1–12)
4th or more child	229 (10)	7 (2–15)	157 (8)	8 (2–17)	72 (19)	4 (0–9)
Type of Delivery						
Vaginal Delivery	2303 (97)	8 (3–17)*	1917 (96)	8 (3–17)*	386 (100)	6 (2–13)
Cesarean Section Delivery	78 (3)	50 (21–93)	78 (4)	50 (21–93)	0 (0)	-
Cost Categories						
Medicine, supplies and procedures	224 (10)	3 (1–8)	181 (10)	3 (1–7)	43 (11)	7 (3–8)
Informal payments	1445 (65)	5 (2–8)	1187 (65)	5 (2–9)	258 (68)	5 (3–8)
Food/baby items	1695 (77)	5 (3–8)	1489 (81)	5 (3–8)	206 (55)	3 (2–8)
Transportation	328 (17)	3 (1–8)	328 (17)	3 (1–8)	–	–

*Wilcoxon-Mann–Whitney test, *p*-value ≤0.05; **Kruskal Wallis test, *p*-value ≤0.05. Column comparisons made

### Who had any OOPE?

Ninety one percent (*n* = 2172) of the sample reported having OOPE; 92 % of JSY beneficiaries and 85 % of women who delivered at home. From the descriptive analysis in Table 1, women who delivered under the JSY program had a significantly higher median, IQR OOPE ($8, 3–18) compared to women who delivered at home ($6, 2–13). The median, IQR OOPE significantly differed between district one ($14, 7–23), and two ($3, 1–7). The median OOPE increased with household wealth for women who delivered in a JSY facility or at home. This pattern was similar in both districts (data not shown). Women from the scheduled tribe caste paid the least OOPE (median $3, IQR 1–6) (Table 1). Women who delivered by caesarean section paid more than six times (median $50, IQR 21–93) the amount compared to women who delivered vaginally (median $8, IQR 3–17).

### Does the JSY cash incentive defray OOPE?

**Fig. 1:**
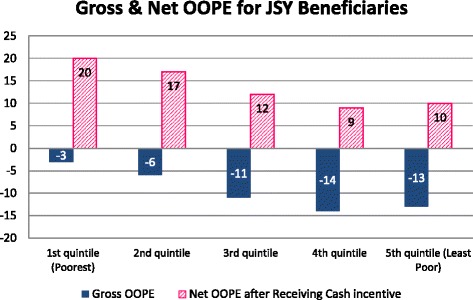
Gross OOPE and Net Gain (gross OOPE minus the incentive $23) for women who delivered in a JSY facility (*n* = 1995), U.S. dollars. Legend: JSY: Janani Suraksha Yojana; OOPE: out-of-pocket expenditures

Among the women who delivered in a JSY public facility, only a quarter (*n* = 504) received the cash incentive upon discharge, 68 % (*n* = 1353) were told to come back to receive the money. Assuming all JSY beneficiaries eventually receive the cash incentive, they would have a median net gain (i.e., JSY incentive – OOPE) of $11. The net gain was larger in district 2 ($19) compared to district 1 ($8) (data not shown). As demonstrated in Fig. 1, women from the poorest wealth quintile had twice the net gain ($20) versus the wealthiest quintile ($10). Only 4 % (21/519) from the poorest quintile incurred OOPE greater than the value of the JSY cash benefit.

### Breakdown of OOPE among JSY and Home deliveries

Among the JSY and home deliveries, 92 % (*n* = 2213) provided disaggregated cost information. Informal payments (64 %) and food/baby items (77 %) were the two most common sources of OOPE (Table 1). The proportion of women incurring OOPE for food and cloth items was higher for JSY beneficiaries (81 %) than for home mothers (55 %). Among the two groups, the median cost for food and baby items was significantly higher ($5) for JSY beneficiaries compared to home deliveries ($3). No other significant differences were found.

**Fig. 2:**
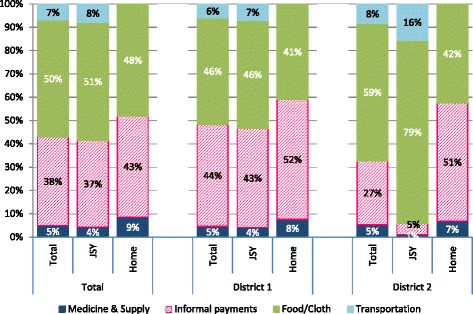
Breakdown of out-of-pocket expenditures by cost categories for JSY beneficiaries and women who delivered at home by district, *n* = 2213*. Legend: JSY: Janani Suraksha Yojana; *This graphs includes only women who were able to provide disaggregated costs (93 %)

OOPE differences by district were found. The breakdown of costs by category was similar for JSY beneficiaries and home deliveries with the exception of the proportion of informal payments in district 2 (Fig. 2). Informal payments constituted only 5 % of the total OOPE for JSY beneficiaries in district 2 compared to 43 % in district 1. The proportion did not differ between districts among home mothers. Among JSY beneficiaries, a higher proportion of wealthy women incurred OOPE as well as had higher median OOPE compared to the poorest quintile.

### Inequalities in OOPE among JSY and home deliveries

**Fig. 3:**
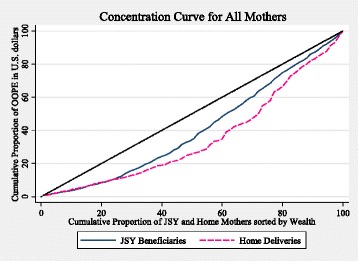
Concentration Curve for JSY beneficiaries and women who delivered at home study. Legend: JSY: Janani Suraksha Yojana; OOPE: out-of-pocket expenditures

Figure 3 displays the concentration curves for OOPE among JSY beneficiaries and home deliveries. Since both curves lie below the line of equality; OOPE for both JSY beneficiaries and home mothers was found to be progressive indicating that poorer households make proportionally less OOP payments during childbirth compared to wealthier households. JSY beneficiaries had less progressive OOPE (CI = 0.189) compared to women who delivered at home (CI = 0.293), however this difference was not significant. The difference was not significant when each district was analyzed separately.

### Impact of JSY on OOPE

When adjusting for confounders, women who delivered in a JSY public facility had 1.58 higher odds (95 % CI: 1.11–2.25) to incur any OOPE than women who delivered at home (Table 2, model 1). Women from district 1 had twice the odds (95 % CI: 1.30–3.18) of having OOPE compared to district 2. Wealth, caste and birth order were not significant predictors of incurring any OOPE.

**Table 2:** Two part model OOPE ($) among women who delivered in a JSY facility (*n* = 1995) and delivered at home (*n* = 386)

Background characteristics	Part 1 of model: AOR *n* = 2381	95 % confidence interval	Part 2 of model: IRR *n* = 2172	95 % confidence interval
Place of delivery				
Home	*1.00*		*1.00*	
JSY (Public Facility)	1.58	(1.11–2.25)*	0.84	(0.73–0.96)*
Districts				
District #2	*1.00*		*1.00*	
District #1	2.03	(1.30–3.18)*	2.36	(2.06–2.69)**
Household wealth				
1st quintile (Poorest)	*1.00*		*1.00*	
2nd quintile	1.49	(0.98–2.27)	1.19	(1.02–1.38)*
3rd quintile	1.53	(0.91–2.56)	1.33	(1.13–1.56)**
4th quintile	1.14	(0.65–1.99)	1.31	(1.11–1.55)**
5th quintile (Least Poor)	1.21	(0.64–2.31)	1.34	(1.02–1.49)*
Caste				
Scheduled Caste (SC)	*1.00*		*1.00*	
Other backward caste (OBC)	1.15	(0.76–1.73)	1.14	(1.01–1.28)*
Scheduled Tribe (ST)	0.97	(0.62–1.52)	0.70	(0.60–0.81)**
General	1.19	(0.63–2.22)	1.07	(0.91–1.26)
Birth order				
1st child	*1.00*		*1.00*	
2nd child	1.21	(0.82–1.79)	0.82	(0.73–0.92)*
3rd child	1.03	(0.63–1.68)	0.71	(0.60–0.83)**
4th or more child	0.64	(0.35–1.17)	0.69	(0.56–0.86)*

Adjusted for age, education and delivery type; *JSY* Janani Suraksha Yojana, *AOR* Adjusted Odds Ratio, *IRR* Incidence Rate Ratio, *p-value ≤0.05; **p-value ≤0.001

However in model 2 among the women who had incurred any OOPE (Table 2), those who delivered under the JSY program paid 16 % less (95 % CI: 0.73–0.96) OOPE than women who delivered at home. Women from district 1 paid more than twice (95 % CI: 2.06–2.69) the OOPE compared to district 2. Increased wealth was also significantly related to higher OOPE. Conversely, being from a scheduled tribe and birth order (women with more children) were related to having lower OOPE.

## Discussion

This is the first study to our knowledge that models predictors of OOPE among JSY beneficiaries and women who deliver at home. We found the large majority of women in our study reported some kind of OOPE. Among the JSY beneficiaries, the poorest women had the largest net gain when the cash transfer was taken into account. Further we found OOP payments under the program were progressive with the most disadvantaged wealth quintiles making proportionally less OOPE compared to wealthier women. Being a JSY beneficiary led to increased odds of incurring OOPE, but at the same time a 16 % decrease in the amount of OOPE incurred compared to women who delivered at home. Finally, wealth was not a predictor of having OOPE, but it was an indicator of how much the women would pay.

In our study, the descriptive and multivariable analysis demonstrated that OOPE in the most vulnerable groups (ST, poorest wealth quintiles and multiparous women) was the smallest. This finding was consistent with another Indian study performed by Mohanty et al. [14] on the District Level Household and Facility Survey (DLHS-3), a national level survey that was conducted in 2007–2008 for births that occurred in the last five years [37]. They also found the amount of OOPE increased with wealth and decreased with birth order. Although there is no universal consensus on why a wealth gradient in OOPE exists, many studies show a positive relationship between income and health expenditures [38]. In other words, the wealthy pay more as they can afford to.

In the descriptive analysis, we found OOPE was higher for women who delivered under the JSY program. However when adjusting for possible confounders, the model showed JSY beneficiaries actually had less OOPE compared to women who delivered at home. Another Indian study that also used data from the DLHS-3 found deliveries conducted under the JSY program at a public facility had higher OOPE compared to home deliveries [15]. This difference was not surprising as only a descriptive analysis was performed and possible confounders were not taken into consideration.

Another probable explanation for these differences is Mohanty et al. reported the amount of OOPE has declined over time for women who delivered in public facilities [14]. Further in 2011, the government of India launched a complementary program to JSY, Janani Shishu Suraksha Karyakram (JSSK), to eliminate OOPE for pregnant women and ensure delivery care was free of cost. Medicines, medical supplies, procedures (surgery, diagnostics and x-rays) food, user fees and referral transport were all supposed to be covered under this program [7].

We found indirect costs (informal payments to staff, food and items purchased for the infant) comprised the largest proportion of maternal expenses. Direct costs (medicines, supplies and delivery services) constituted the smallest share of spending among both JSY beneficiaries (4 %) and women who delivered at home (9 %). Conversely, Skordis-Worral et al. [23] found in urban Indian slums that direct medical costs were responsible for the majority of OOPE. Context is extremely important when interpreting results for studies performed in India given the heterogeneity between different parts of the country and stark differences between rural and urban areas. Our study was performed in a rural setting, the poor in urban and rural settings experience different kinds of access issues possibly explaining some of the difference in results.

### Free delivery under the JSY program?

High OOPE are a well-known constraint to the utilization of delivery services where ready access to cash is not available for many rural households, especially the poor whose ability to pay can be temporal or mainly rely on seasonal production like farms [39]. The vast majority of women in our study incurred some amount of OOPE. While these women paid less as JSY beneficiaries compared to women who delivered at home, they did not have a free delivery as intended by the public health care system. As mentioned above, in this study the direct medical costs were not the driving force behind OOPE, but informal payments to the staff. A qualitative study from the same area found women knew they were not supposed to make payments to the staff but did anyway out of fear of not receiving appropriate care in a timely manner [40].

Although from the perspective of the Indian government, the primary purpose of the cash transfer is to incentivize women for a free delivery in a public health facility and *not* to cover OOPE, the same qualitative study found many women do in fact intend to use it to compensate for the OOPE incurred [40]. Only 25 % of the women who delivered in a JSY public facility in our study received the cash incentive upon discharge. While a previous study that took place in the same area found 85 % of the women received the benefit within two weeks of delivery [41], if the cash benefit is expected by the women to cover their OOPE, it needs to be received upon discharge. These bureaucratic issues related to receiving the JSY cash incentive and informal payments to public health facility staff members undermine the program and could potentially cause future uptake problems if not addressed.

### Does JSY reduce financial access barriers?

Population based surveys have shown a significant proportion of women report cost as the major barrier to an institutional delivery, [2, 37] and other research reports support this [10, 23, 42]. However, our model showed JSY beneficiaries had lower OOPE compared to women who delivered at home. In addition, it is important to note the JSY beneficiaries in our study received a net gain, especially among the poorest quintiles, after receiving the cash incentive ($23). Although this is to be expected since the poorest quintiles paid the least, regardless this would imply the program is reaching and assisting the most vulnerable groups. In our setting the incentive was more than adequate to cover the OOPE, while an earlier study from Orissa found the magnitude of the cash incentive was not large enough to compensate for the entire OOPE amount [43]. Another Indian study reported the JSY program provided women with some financial protection, though it was limited and did not cover the entire sum [14].

Further, the women in our study who delivered at home did not cite financial barriers as the justification for a home delivery. A recent qualitative study from the same area also found that cost was not a deterrent for most of the study participants who delivered at home [40]. This implies other access barriers persist, some of which may be remedied but not necessarily by a cash transfer.

### JSY role in reducing OOPE inequalities and inequities: wealth quintiles and OOPE in the JSY

Our concentration curves showed that the OOPE for women who delivered under the JSY program was pro-poor; poorer households made proportionally less OOP payments during childbirth compared to wealthier households (i.e., a progressive system). From a strict equality perspective, the distribution was not equal. However, some would argue the wealthier households have the means to pay for services while the poorer ones do not [44]. The OOPE may not be equal; nevertheless the program is making OOPE more equitable and fair.

In general, health care financing systems that are progressive tend to have a redistributive nature. Taxes, where wealthier households pay higher amounts of money compared to poorer ones, is one example. Social service provision by the government (e.g., offering free delivery care) is another [44]. So while vulnerable groups often have more healthcare needs, despite contributing less, they are able to obtain the same service as their wealthier counterparts. Yet, it is unknown whether the poorest women are in fact acquiring the same level of service. In our study, when comparing the prevalence of different costs and the median expenditure between the poorest and wealthiest quintiles of JSY mothers, it appears there is a higher prevalence of informal payments among the wealthiest quintile and higher amounts are paid. This may just reflect the fact wealthier women have more disposable income to spend or do wealthier women informally pay for better services even within the same facilities? A program like JSY has the power to promote equality and equity in access to delivery care, and while several argue the overall quality of care administered in JSY public facilities is low, [45–49] it is still important to ensure all receive the best care possible regardless of whether they have the means to pay for it.

We have presented differences in OOPE between the two study districts throughout the paper. In district 1, a woman is more likely to have an OOPE and pay higher amounts. Since the OOPE amount was higher in district 1, JSY beneficiaries’ net gain was also smaller. These findings were not surprising considering the heterogeneity between the districts. The women are much poorer in district 2 compared to district 1 so this would have an impact on the amount of money they spent. This also has an implication for the relative worth of the incentive to women in their respective districts. Another reason could be attributed to the sampling methodology. While district 1 included all blocks, district 2 only included 2 of the 5.

## Methodological considerations

Though there have been some studies assessing the OOPE experienced during childbirth in India, few have looked at this from an equity perspective under the JSY program. The previous studies have used secondary data that did not allow for cost disaggregation, assessed a time period when JSY coverage and overall institutional delivery was low or were small in size [13–17, 23]. Furthermore, in some studies the costs were collected for deliveries that occurred in the previous five years, thus probably suffered from substantial recall bias. Considering how quickly the JSY program has increased institutional birth proportions in such a short period of time, it is important to have recent data that reflects the current situation.

Reports have found in many Asian countries that families borrow money to pay for maternal related costs thus being forced to forego essential items like food and education to repay the loans. These costs have a ripple effect on the family for years to come [50]. We did not enquire about the financing sources used to pay for the hospital delivery costs. This limits our ability to understand the role of JSY in providing financial protection and reducing subsequent impoverishment. So while this study has shown JSY beneficiaries have reduced OOPE compared to women who delivered at home, further research is needed to understand the magnitude of the reduction in relation to the family’s overall poverty status.

Many studies highlight the limitations (e.g., recall bias and over/underreporting) associated with collecting health expenditure data [51–53]. Cost data was collected shortly after delivery and triangulated with other family members to minimize recall bias. A disaggregated cost collection design was used to improve accuracy and avoid underreporting of expenditures. While our study design minimized recall bias as the study participants were interviewed within a week of delivery, we do need to acknowledge the possibility of underreporting for women who were interviewed in a health facility because of staff presence.

The sex of the infant has been reported in the literature as a determinant of OOPE [14]. Data was not collected on the sex of the infant, therefore we could not adjust for it in the model. It is reasonable to assume in this setting the infant’s sex would influence OOPE, it is well documented that families make higher informal payments when a male child is born [14]. This could affect the precision of the analysis and the significance of other explanatory variables could be overestimated. However, we do not have any reason to think that the sex of the infant is differently distributed among the two groups.

There could be an argument for the payments made to the *dai* to be considered as a formal payment and classified as payment for ‘medicines, supplies and procedures’. However we chose to classify these payments as informal because (a) the *dai* is not formally trained, (b) is not a part of the formal health system and (c) remuneration to the *dai* is negotiable i.e., there are not fixed stipulated fees, she is remunerated often partly in cash and partly in kind based on the ability of the mothers family to pay and their relationship with the *dai*.

Sampling: The districts were sampled in the exact same way with the exception of the number of blocks chosen. However, the process of how the women were selected for the study was the same in both districts. As the districts in the state and in the rest of the country are very heterogeneous, generalizability of our results needs to be done with caution.

## Conclusion

OOPE is still prevalent among women who deliver under the JSY program as well in home deliveries. In JSY, OOPE varies by income quintile; the wealthier women pay more OOPE. There is a net gain for all quintiles when the incentive is taken into consideration, highest gains occur for the poorest women. OOPE was largely due to indirect costs like informal payments, food and cloth items for the baby and not direct medical payments. Further, we found OOP payments under the program were progressive with the most disadvantaged wealth quintile making proportionally less OOPE compared to wealthier women. Being a JSY beneficiary led to increased odds of incurring OOPE, but at the same time a decrease in the amount of OOPE incurred compared to women who delivered at home. While wealth was not a predictor of having OOPE, it was an indicator of how much the women would pay. The program seems to be effective in providing financial protection for the most vulnerable groups (i.e., women from poorer households and disadvantaged castes).

## Ethical considerations

Ethical approval for the study was granted by the Ethics Committee of R.D. Gardi Medical College (Ujjain, India) and Karolinska Institutet (Stockholm, Sweden), reference # 2010/1671–31/5.

## Abbreviations

AOR: adjusted odds ratio
CI: concentration index
DLHS: District Level Household and Facility Survey
GLM: generalized linear model
GOI: Government of India
INR: Indian rupees
IQR: interquartile range
IRR: incidence rate ratio
JSSK: Janani Shishu Suraksha Karyakram
JSY: Janani Suraksha Yojana
MMR: maternal mortality ratio
MP: Madhya Pradesh
OBC: other backward caste
OOPE: out-of-pocket expenditures
SC: scheduled caste
ST: scheduled tribe
US$: U.S. dollar
VIF: variance inflation factor

## Footnotes

1 Caste is a historical form of social stratification. Although the use of castes is now outlawed, social inequalities and discrimination continue to persist as a result of it. Other backward caste, scheduled caste and tribe are groups of people historically subjected to social disadvantage. They were awarded special status by the Constitution of India that entitles them to specific social benefits.
